# ADME prediction with KNIME: A retrospective contribution to the second “Solubility Challenge”

**DOI:** 10.5599/admet.979

**Published:** 2021-07-12

**Authors:** Gabriela Falcón-Cano, Christophe Molina, Miguel Ángel Cabrera-Pérez

**Affiliations:** 1 Unit of Modelling and Experimental Biopharmaceutics. Centro de Bioactivos Químicos. Universidad Central “Marta Abreu” de las Villas. Santa Clara 54830, Villa Clara, Cuba; 2 PIKAÏROS S.A., 31650 Saint Orens de Gameville, France; 3 Department of Pharmacy and Pharmaceutical Technology, University of Valencia, Burjassot 46100, Valencia, Spain; 4 Department of Engineering, Area of Pharmacy and Pharmaceutical Technology, Miguel Hernández University, 03550 Sant Joan d’Alacant, Alicante, Spain

**Keywords:** Second Solubility Challenge, Quantitative Structure-Property Relationship (QSPR), KNIME, aqueous solubility, ADME, machine learning, Random Forest, supervised recursive variable selection

## Abstract

Computational models for predicting aqueous solubility from the molecular structure represent a promising strategy from the perspective of drug design and discovery. Since the first “Solubility Challenge”, these initiatives have marked the state-of-art of the modelling algorithms used to predict drug solubility. In this regard, the quality of the input experimental data and its influence on model performance has been frequently discussed. In our previous study, we developed a computational model for aqueous solubility based on recursive random forest approaches. The aim of the current commentary is to analyse the performance of this already trained predictive model on the molecules of the second “Solubility Challenge”. Even when our training set has inconsistencies related to the pH, solid form and temperature conditions of the solubility measurements, the model was able to predict the two sets from the second “Solubility Challenge” with statistics comparable to those of the top ranked models. Finally, we provided a KNIME automated workflow to predict aqueous solubility of new drug candidates, during the early stages of drug discovery and development, for ensuring the applicability and reproducibility of our model.

## Introduction

Pharmacokinetic parameters are usually influenced by a combination of different physicochemical properties. Among these, solubility has occupied a very important role due to its influence on the absorption process. The need to balance solubility, avoiding excess or insufficiency, is a challenge from the perspective of drug discovery.

In this regard, several research efforts have been made to provide accurate prediction of aqueous solubility through Quantitative Structure-Property Relationship (QSPR) approaches. Undoubtedly, the first and second “Solubility Challenges” proposed by Llinas et al. have been a very effective indicator of the progress and state-of-art of solubility estimation [1,2]. Recently, Llinas et al. have reviewed the results of the second “Solubility Challenge” to analyse the evolution of the computational methods used in this prediction task and the influence of data quality on the results [3].

In our previous publication, we presented a new method based on recursive random forest approaches to predict aqueous solubility values of drug and drug-like molecules [4]. It was based on the development of two novel recursive machine-learning approaches used for data cleaning and variable selection, and a consensus model generated by the combination of regression and classification algorithms. This model was able to provide good solubility prediction compared to many of the models described in the literature. Considering that our model was developed from a database of aqueous solubility values with limited information on the experimental conditions of the solubility assay, could our model successfully predict the intrinsic solubility values of the two sets of drugs used in the second “Solubility Challenge”?

The present study describes the performance of our model with the molecules of the second “Solubility Challenge” and the comparison of the results with those obtained with the best performing models of the competition. It is necessary to clarify that, for this task, the model was not trained, retrained or optimized based on the molecules of the challenge tests, i.e., the model parameters or hyper-parameters remained exactly the same as those set in previously published work [4].

## Materials and methods

### Challenge sets

The second “Solubility Challenge” consisted of evaluating the intrinsic solubility estimation of two sets of drugs. The first set is composed of 100 drugs with an average inter-laboratory standard deviation estimated of ~0.17 log units. The second test set consists of 32 “difficult” drugs, characterized by poor inter-laboratory reproducibility: Standard Deviation ~0.62 log units. A detailed list of these molecules have been shown in a previous paper [3].

### Software

The Konstanz Information Miner (KNIME) is a free and public software tool that has become one of the main analytical platforms for innovation, data mining and machine learning. The flexibility of workflows developed in KNIME to include different tools allows users to read, create, edit, train and test machine learning models, greatly facilitating the automation of predictions and application by any user [5,6]. In this study, we used the open source software KNIME Analytical Platform version 4.0.2 [7] and its free complementary extensions for transformation, analysis, modelling, data visualization and data prediction. For the generation of molecular descriptors from structures, the “Descriptor” node from “alvaDesc” extension [8] and the “RDKit Descriptor” node [9] were employed.

### Modelling dataset

To predict the molecules of the second “Solubility Challenge”, we used as the training set the curated set of aqueous solubility published in our previous paper. This set consists of two large aqueous solubility databases [10,11]. For each molecule, taking the SMILES (Simplified Molecular Input Line Entry Specification) code as input format, a structure cleaning, standardization, and duplicate removal protocol was developed. The InChi (IUPAC International Chemical Identifier) code was used for duplicate identification and the standard deviation among experimental measurements was computed. A detailed description of this procedure has been shown in our previous article [4]. Although the hypothesis that -*the quality of the experimental data is the main limiting factor in predicting aqueous solubility*- has been challenged [12], any variability in the experimental protocol is always “noise” for *in silico* modelling purposes. In this sense, our model had several challenges such as: 1) the pH value for the solubility measurement of the collected compounds was not stated, 2) the solid form of the molecule (polymorphs, hydrates, solvates, amorphous) was not characterized in the reported solubility measurements, 3) it was not possible to verify the type of solubility measurement (kinetic or thermodynamic) and 4) the experimental measurement method was not specified.

### Modelling algorithm

Due to the uncertainty of the database, we considered the importance of a rigorous protocol for data selection in the development of the original model, in order to discriminate those molecules with potential unreliability. As a first step, we selected a RELIABLE Test Set, consisting of molecules with more than one reported measurement and with inter-source standard deviation greater than 0 and less than 1 logarithmic unit. We used beyond 1 logarithmic unit as a threshold to discriminate unreliable samples. This RELIABLE Test Set was used for model optimization.

From the QSPR perspective, it is necessary to select a set of descriptors that leads to the most predictive model and facilitates model interpretation. To this end, we developed a recursive variable selection algorithm based on regression random forest (RRF). RRF is a widely used ensemble method that assembles multiple decision trees and outputs the consensus predictions from individual trees [13]. It is recognized for its ability to select “important” descriptors. Based on this ability, we use the number of occurrences of a variable in the RRF as a measure of the descriptor’s importance, combined with a correlation analysis between variables to avoid collinearity. Each numerical descriptor was injected in the RRF in two ways: non-shuffled and shuffled. Once the individual decision trees were trained and extracted from the ensemble, the total number of occurrences of each variable was calculated. Only variables with a number of occurrences greater than a marginal threshold of 110 were retained. Among those, variables were discarded if the non-shuffled variable had a number of occurrences lower than the number of occurrences of its homologous shuffled variable. All shuffled variables were eventually discarded too. The final set of variables was selected recursively by initially computing the linear correlation between variables, and then keeping only those with the highest number of occurrences among variables with a correlation coefficient greater than a threshold of 0.51 between them.

In an attempt to reduce the uncertainty of the data, independent of any external set, a cleaning procedure based on an RRF approach was developed. This procedure uses the Prediction Variance (PV) of the RRF as a metric to discriminate unreliable samples. The PV is an RRF score that highlights the variability of each individual prediction with respect to the mean. A high PV can be a sign of anomalous behaviour or uncertainty. This procedure was applied to the UNRELIABLE Set, i.e. molecules with aqueous solubility standard deviation between sources equal to 0 or greater than 1. To set the parameters of this algorithm, the minimization of the root mean squared error (RMSE) of the RELIABLE Test was used as the objective function. First, the UNRELIABLE Set was randomly divided into two sets of 50 % and 50 % cardinal. A regression random forest was trained on one of the two sets and used to predict the aqueous solubility and PV of the other set. In addition, the PV of the out-of-bag samples was also calculated. Recursively, molecules were classified as within the PV threshold (CLEAN data) or alternatively as beyond the PV threshold (UNCLEAN data), until no molecules changed from CLEAN to UNCLEAN labelled set or vice versa.

Using the CLEAN set, a Gradient Boosting Model (GBM) was trained for classification using logS = -2 as the cut-off to label molecules into highly soluble or soluble and slightly soluble or insoluble. Two independent RRF models were developed based on these two subsets of labelled molecules and one more RRF model was trained on all CLEAN data. Finally, the average prediction among the three GBM models was assumed as the final prediction value. The parameters of all models were optimized based on the RMSE minimization of the RELIABLE test set. Full details on our developed algorithm are given in previous published paper [4].

### Second “Solubility Challenge” prediction

First, we ensured that all test set molecules found in the initial source set used as the training set were removed. Since the model was previously validated using the RELIABLE Test Set and by 5-fold cross-validation, we used the entire database (including the RELIABLE Test Set) to predict the test challenge samples. To analyse the performance of the solubility regression models, two types of coefficient of determination (r^2^), root mean squared error (RMSE), mean absolute error (MAE), bias and the percent of molecules with an absolute error less than 0.5 logarithmic units (% 0.5 log) were calculated.

## Results and Discussion

### Model performance

**Figure 1.**
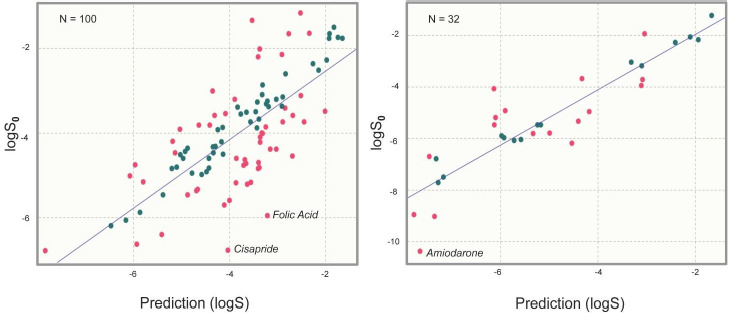
Plot of log *S* (predicted) vs log *S*_0_ (experimental) for both test sets. Molecules with residual values higher than 0.5 (logarithm units) are highlighted in red.

The statistics obtained for both sets (Test Set 1 = 100 molecules and Test Set 2 = 32 molecules) are shown in Table 1 and Figure 1. To demonstrate model robustness, the results are reported as mean and standard deviation (Std).

**Figure 2.**
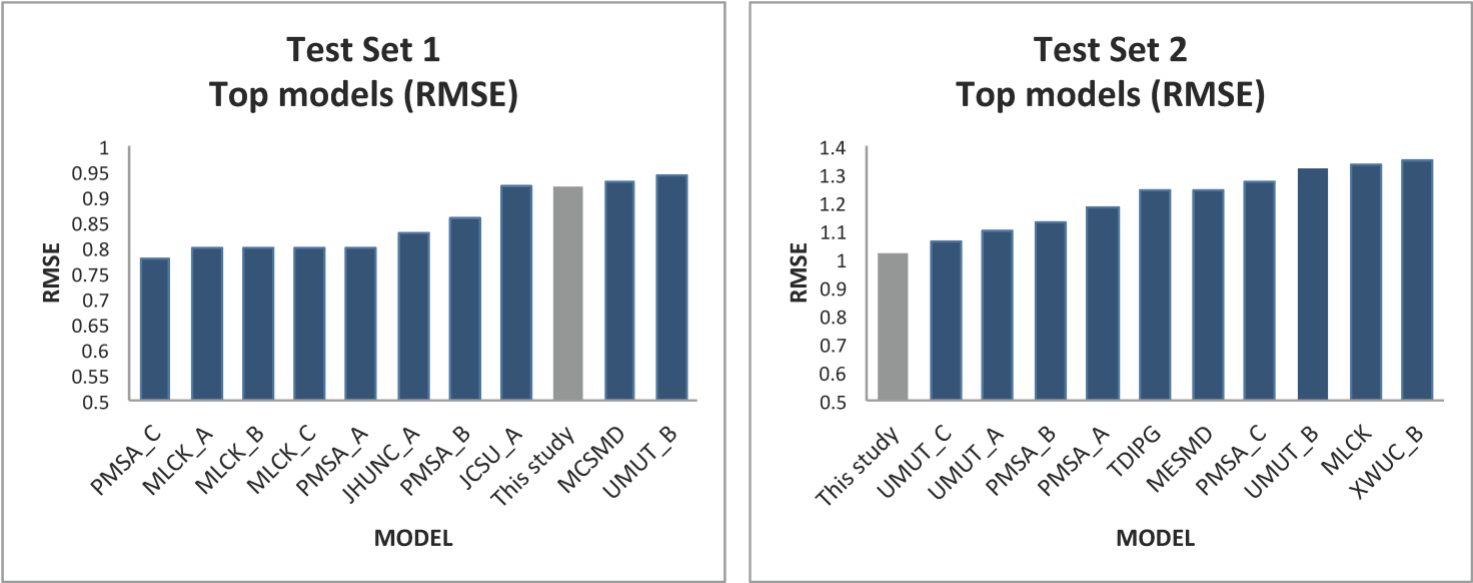
Comparison between the top-rank models of the Second Solubility Challenge and our results (according to RMSE)

Figure 2 compares our results with the top-rank models of the second “Solubility Challenge”. According to the mean RMSE value, our consensus model ranks ninth among the top-ranked models for the prediction of Test Set 1 and first for the prediction of Test Set 2.

Although there are no significant differences in terms of prediction performance, the training set we have used contains aqueous solubility measurements under non-specified experimental conditions (pH, method and solid form), without information on their type of solubility (aqueous or intrinsic). It is known that the presence of acidic and basic groups in a molecule and the pH of the medium affect the solubility value. Intrinsic solubility corresponds to the solubility of the uncharged molecular species, whereas aqueous solubility depends on the pH used for measurements. Therefore, not all the values in the training set are true intrinsic solubility values, which influences the model prediction of the external test set with intrinsic solubility measurements, leading in some cases to higher uncertainty for samples contained in the training set.

**Figure 3.**
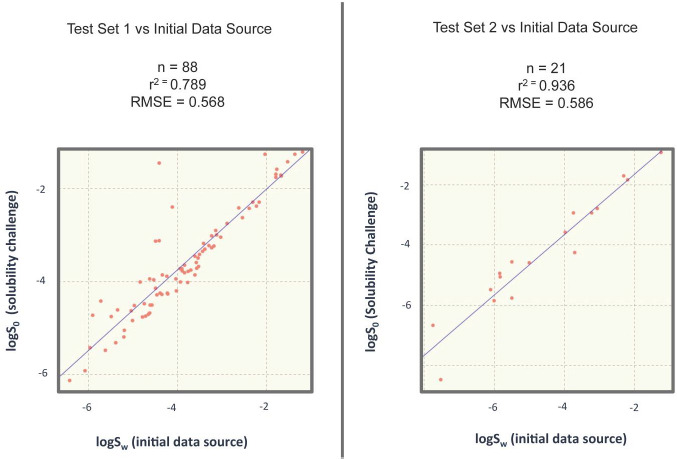
Overlapping log *S*_0_ against log *S*_w_ analysis between the molecules of the second “Solubility Challenge” and the training set. For modelling purposes, these overlapping molecules were eliminated from the training set.

We analysed the overlap of our source set with the molecules from the second “Solubility Challenge”, resulting on two overlaps of 88 and 21 molecules, 1^st^ and 2^nd^ test respectively. Only for the case of these 109 overlapping molecules, a correlation analysis was performed between the intrinsic solubility values reported in the second “Solubility Challenge” and the aqueous solubility values reported in our initial source set. The overlapping molecules were eliminated from the training set for modelling purposes. This analysis is shown in Figure 3.

Considering the lack of real intrinsic solubility values in the training set, the most problematic molecules in the second “Solubility Challenge” should be the ionizable compounds. The analysis of residuals showed that Amiodarone (TS2), Cisapride (TS1) and Folic Acid (TS1) are response outliers. All of them contain at least one acidic or basic functional group and are practically insoluble compounds. For these molecules, the aqueous solubility value (log *S*_w_) is different from the intrinsic solubility value, since not enough solute is dissolved to modify the pH in order to maintain a near-neutral species in the poorly buffered medium. Table 2 describes the values of log *S*_0_ (second “Solubility Challenge”), log *S*_w_ (initial data source), log *S*_w_ (reported in other sources) and log *S*_w_ (predicted).

To assess whether the method was able to deal with the uncertainty in the data, a simple experiment was performed. As shown in Figure 3, 88 molecules from the first test set of the challenge overlapped with our initial source set. A correlation analysis between the two solubility values reported by each overlapping molecule showed a root mean squared error of 0.568 log units. We assume that the value reported in the challenge refers to a curated and reliable measurement, whereas the value reported in our initial source set could be of potential uncertainty. There is a significant difference between the two sets of values for the 88 molecules (Confidence interval (CI): 95 %; p = 2.9E-5). Next, a paired-sample t-test was developed for comparing the performance of two models based on two different training sets: (a) the literature solubility data reported in our initial source set and (b) the reliable intrinsic solubility measurements reported in the first set of the challenge. Both models were evaluated on the second challenge test. There was no significant difference (CI: 95%, p = 0.58) between the root mean squared errors achieved on the second challenge test using one or the other training sets. However, if a single random forest regression without recursive selection of data and variables and without applying a consensus model is used as the modelling algorithm, the *t*-test highlights a significant difference (CI: 95%; p = 3.3 E-6). The influence of data quality on model performance depends on the modelling procedure used. Thus, data quality was not the determinant factor when an appropriate modelling approach was designed to address data uncertainty by selecting the most important variables and using a consensus model of combined single model predictions. Table 3 shows a review of the results.

### Automated system for aqueous solubility prediction

We trust there is a need to make publicly available a reliable and diverse data set of intrinsic solubility measurements for a rigorous comparison between modelling algorithms, due to the relative influence of data quality on the performance of a model. Furthermore, applicability and reproducibility of solubility QSPR models should be a priority for data to be **F**indable, **A**ccessible, **I**nteroperable and **R**eusable (FAIR) [16–18]. In this regard, the final purpose of the current commentary is to make publicly available an automated system for *in silico* aqueous solubility assessment. Our model has been successfully validated in a previous published study and has been blind tested with the second “Solubility Challenge”, showing an adequate performance. The KNIME workflow published with the paper contains the results of our model on the second “Solubility Challenge” and allows the prediction of new sets. The user can download the workflow and follow the instructions it contains from https://pikairos.eu/download/aqueous_solubility_prediction/. We developed a version based on RDKit and AlvaDesC descriptors, calculated using the “Descriptor” node contained in the “alvaDesc” extension. AlvaDesc 1.0.16 is available with academic or commercial licenses, which can be obtained by requesting a quote online (registration required) or by contacting them directly by email (chm@kode-solutions.net). Only the SMILES codes of the structures are needed for aqueous solubility prediction, as the model does not require any experimentally determined value for solubility calculation. The model is characterized by its simplicity since it is only based on 0-2D descriptors. In addition, the model is implemented in the open-source analytics platform KNIME, which is a user-friendly software suitable for further data analysis and visualization.

## Conclusions

The results obtained with the evaluation of the second “Solubility Challenge” reinforce the idea that data quality is not the major limiting factor for obtaining adequate solubility predictions if the implemented modelling methodology can cope with data uncertainty. In our case, the developed algorithm was able to overcome data variability to obtain acceptable aqueous solubility prediction results. The results published here are a blind prediction, since the experimental aqueous solubility values of the challenge test set were not accessible at the time of our model development and training. Although the achieved performance is comparable to those reported in the review of the second Solubility Challenge, our model is only based on public data compared to some of the best models of the second Solubility Challenge, which were based on the huge aqueous solubility databases available from pharmaceutical companies. Furthermore, the algorithm of our model is global, as demonstrated by the use of generic data without the bias of “training close to the test data”. The automation of the proposed methodology and its possible application on larger databases, collected under more homogeneous conditions, could be a step forward to improve solubility prediction during drug discovery and development stages. In attention to the importance of sharing data and methods to ensure reproducibility and applicability of QSPR models, we made the data publicly available along with our predictive model based on the KNIME Analytical Platform as a new free tool for the assessment of aqueous solubility of drug candidates.

## Figures and Tables

**Table 1.** Performance of the final consensus model for the molecules of the second “Solubility Challenge”

Test	r^2^ (validation)		r^2^ (Pearson)		RMSE (validation)		MAE (validation)		Bias		% 0.5 log	
	Mean	Std	Mean	Std	Mean	Std	Mean	Std	Mean	Std	Mean	Std
Test Set 1 (N = 100)	**0.458**	0.01	0.58	0.01	**0.925**	0.03	0.74	0.03	-0.234	0.01	40	1
Test Set 2 (N = 32)	**0.777**	0.02	0.78	0.01	**1.019**	0.1	0.77	0.1	-0.278	0.02	40	6

**Table 2.** Summary of solubility values for the outliers

Structure	Name	log *S*_0_^a^	log *S*_w_^b^ (initial source set)	log *S*_w_ (predicted)	log S_w_^c^ (other sources)
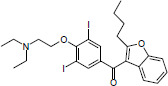	Amiodarone	-10.4	-9.35	-7.54	-7.17 [14]
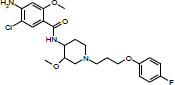	Cisapride	-6.78	-5.23	-4.27	-4.7 [15]
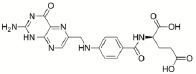	Folic Acid	-5.96	-5.44	-3.12	> -2.87 [15]

^a^Intrinsic Aqueous Solubility reported in the second “Solubility Challenge”

^b^Aqueous Solubility reported for the three outliers in the initial source set

^c^Aqueous Solubility reported in other sources

**Table 3.** Mean with Std statistics based on two training sets when predicting the second test of the second “Solubility Challenge” using our method (Recursive Random Forest (consensus)) versus a single RRF: reliable solubility measurements (data challenge) and literature solubility data.

Test	Reliable solubility measurements (data challenge) n (training) = 88		Literature solubility data (reported in Initial Data Source) n (training) = 88	
	r^2^ (validation)*	RMSE (validation)*	r^2^ (validation)*	RMSE (validation)*
Recursive Random Forest (consensus)	0.30 (0.05)	1.79 (0.06)	0.29 (0.05)	1.80 (0.05)
Single Random Forest Regression	0.19 (0.01)	1.93 (0.02)	0.14 (0.06)	1.98 (0.06)

*The results are reported as Mean (Std). The Std was computed by repeating 10-times the modelling procedure.

## Abbreviations

ADME: Absorption-Distribution-Metabolism-Excretion
QSPR: Quantitative Structure-Property Relationship
KNIME: Konstanz Information Miner
RF: Random Forest
RRF: Regression Random Forest
Std: standard deviation
y_*i*_^obs^: experimental intrinsic solubility value
y*_i_^calc^*: predicted aqueous solubility value (model)
r^2^ (val): the square of the correlation coefficient of regression (validation). r^2^ (val) = r^2^ = 1 - Σ_i_ (y*_i_^obs^* - y*_i_^calc^*)^2^ / Σ_i_ (y*_i_^obs^* - <y^obs^>)^2^, where y*_i_^obs^* is the experimental log *S*_0_ and <y^*obs*^> is the mean value of the experimental log *S*_0_ values.
r^2^ (Pearson): the square of the correlation coefficient of regression (Pearson). r^2^ (Pearson) = r^2^ = 1 - Σ_i_ (y*_i_^obs^* - *a b*y*_i_^calc^*)^2^ / Σ_i_ (y*_i_^obs^* - <y^*obs*^>)^2^, where y*_i_^obs^* is the experimental log *S*_0_, <y^*obs*^> is the mean value of the experimental log S_0_ values, *a* is the intercept and *b* the slope.
RMSE: the root mean squared error. RMSE = 1/n Σ_i_ (y*_i_^obs^* – y*_i_^calc^*) ^2^]^1/2^, where y^*obs*^/ y^*calc*^ = observed/calculated value of logS_0_, n = number of samples.
MAE: mean absolute error. MAE = 1/n Σ_i_ |y*_i_^obs^* – y_i_^calc^|, where y^*obs*^/ y^*calc*^ = observed/calculated value of log *S*_0_, n = number of samples. Bias = 1/n Σ_i_ (y*_i_^obs^* – y_i_^calc^), where y^*obs*^/ y^*calc*^ = observed/calculated value of log *S*_0_, n = number of samples.
TS: Test Set
PVS: Prediction Solubility Variance
CI: Confidence interval
FAIR: **F**indable, **A**ccessible, **I**nteroperable and **R**eusable
